# Investigation of Wettability, Thermal Stability, and Solar Behavior of Composite Films Based on Thermoplastic Polyurethane and Barium Titanate Nanoparticles

**DOI:** 10.3390/polym16233259

**Published:** 2024-11-23

**Authors:** Dilayda Kanmaz, Rumeysa Celen, Esra Karaca, Gizem Manasoglu

**Affiliations:** 1 Department of Textile Engineering, Engineering Faculty, Bursa Uludag University, Bursa 16059, Turkey; dilaydakanmaz@uludag.edu.tr (D.K.); ekaraca@uludag.edu.tr (E.K.); gmanas@uludag.edu.tr (G.M.); 2 Department of Biomaterials, Graduate School of Natural and Applied Sciences, Bursa Uludag University, Bursa 16059, Turkey

**Keywords:** barium titanate, thermoplastic polyurethane, solution casting method, composite films, wettability, solar properties

## Abstract

Herein, composite films were produced by incorporating different amounts (1, 3, 5, and 7%) of barium titanate nanoparticles into the thermoplastic polyurethane matrix using a solution casting method. This study examined the impact of the presence and concentration of a barium titanate additive on morphologic properties, mechanical performance, thermal stability, solar behavior, and wettability of produced film samples. The films were characterized by Fourier transform infrared spectroscopy, differential scanning calorimetry, thermal gravimetric analysis, scanning electron microscope, ultraviolet-visible near-infrared spectrophotometer, water contact angle, and tensile strength measurements. In the present study, the mass loss of samples containing 7% barium titanate was 24% lower than that of the pure polyurethane reference. The increase of barium titanate rate added to polyurethane enhanced the solar reflectance property of the films, including the near-infrared region. As a prominent result, the transmittance value decreased significantly compared to the reference in the ultraviolet region, and it dropped to 3% for the highest additive concentration. The contact angle values of polyurethane films increased by 11–40% depending on the barium titanate addition ratio. The nano additive also positively affected the mechanical performance of the reference polyurethane film by slightly increasing the tensile strength values.

## 1. Introduction

In the past few years, there has been a significant increase in scientific and technological research on composites made of polymers and inorganic materials [1]. The polymer/inorganic nanocomposites are a type of composite material that consists of nanometer-sized inorganic nanoparticles uniformly dispersed in and fixed to a polymer matrix [2,3]. Combining the unique properties of both polymers and inorganic nanoparticles leads to the production of novel high-performance materials that find applications in many industrial fields [1].

Various methods are commonly used to prepare polymer-based composite films, including solution casting, compression molding, and extrusion [4]. The solution casting method is one of the most widely used and practical methods in composite film production [5]. This method consists of four steps: dispersing additives in the polymer solution, pouring the mixture into a mold or film applicator, evaporating the solvent, and separating the film from the cast surface [6]. The solution casting method is easy to use, controllable, and cost-effective on a laboratory scale [7,8,9]. At the same time, the solution casting method offers advantages such as porosity, sufficient pore size, and controllable film thickness (with the gap between the substrate and the film applicator) [8,10].

Thermoplastic polyurethanes (TPUs) consist of linear block copolymers that have both hard and soft segments [11]. Thanks to their particular chemical structure, TPU is indispensably used as a matrix material in various applications, such as the automobile, aerospace, and clothing industries, as well as in buildings [11,12,13,14]. However, there is still a gap in the literature for improving specific properties of TPUs by including nanometric fillers.

Barium titanate (BT) is an inorganic compound with the chemical formula BaTiO_3_ [15]. For over six decades, BT has been of great interest due to its attractive properties. Firstly, it is highly stable with excellent mechanical, electrical, and chemical properties. Secondly, since it is a lead-free material, it does not threaten the environment and human health, unlike other ceramic materials [16]. Finally, BT possesses a high refractive index of 2.40 [17], indicating that it is a competitive candidate for solar reflection. In the literature, barium titanate was added to different structures such as fibers/yarns [18,19,20,21,22,23,24,25,26], fabrics [27,28,29], composites [30,31,32,33,34,35,36], and films [37,38,39,40,41,42,43] to improve their dielectric, ferroelectric, optoelectric, and electromagnetic shielding properties. 

Numerous inorganic particles such as antimony trioxide (Sb_2_O_3_) [44,45], strontium titanate (SrTiO_3_) [46], barium titanate (BaTiO_3_) [30], and zinc sulfide (ZnS) are capable of significantly enhancing the solar reflectivity of polymers. Solar radiation energy is primarily composed of 5% UV light, 43% visible light, and 52% near-infrared light. Ideally, the reflectivity of all three bands should be increased to maximize solar reflectivity [47]. Materials with a high refractive index represent excellent scattering ability [44,48], and usually possess higher reflectance [49,50]. To prepare composite materials with high solar reflection, it is possible to introduce inorganic materials with high refractive index into the polymer matrix.

To our knowledge, some studies in the literature have explored the addition of barium titanate to TPU composite films. In one of these, Şen et al. examined the thermal stability, mechanical properties, and hydrophobicity properties of barium titanate/TPU composite films [51]. Seo et al. prepared a nanocomposite film of waterborne polyurethane (WPU) reinforced with barium titanate nanoparticles and focused on its piezoelectric properties [52]. In another study, Baidya et al. developed and characterized castor oil-based PU/barium titanate composite coating films and examined their dielectric and piezoelectric properties [53]. There have been no reports on the solar properties of the barium titanate nanoparticles added to TPU films to date. The current study focused on this aspect of films, such as reflectance, transmittance, absorbance, and opacity, as well as examining the strength, thermal, morphological, and water-repellency properties of barium titanate-added polyurethane films. Nanocomposite TPU films were produced using the solution casting method, with a barium titanate additive concentration ranging from 1% to 7%. As the barium titanate amount increased, the targeted properties of polyurethane films improved. 

The demand for polyurethanes as high-performance materials in various industries is increasing daily [54]. TPU films produced in the study, which demonstrate enhanced properties when combined with a barium titanate additive, are believed to have diverse applications. For instance, their improved thermal stability makes them suitable for high-temperature applications and thermal management systems [54,55]. Additionally, their enhanced hydrophobic characteristics allow them to serve as effective coating materials in highly corrosive environments [56]. Furthermore, these films can be utilized in ultraviolet protection and cooling applications due to their ultraviolet absorbance and solar reflectance properties [57]. Although the electrical properties of the composite films were not investigated in this study, they are thought to be open to development for possible use in areas such as solar cells, ceramic capacitors, and electro-optical devices due to the known high piezoelectric and dielectric properties of barium titanate [58,59,60].

## 2. Materials and Methods

### 2.1. Material

Elastollan^®^ commercial thermoplastic polyurethane with a molecular weight of 107,010 g/mol was obtained from BASF (Ludwigshafen, Germany). N,N′-dimethylformamide. (DMF) was supplied from Sigma-Aldrich (St. Louis, MO, USA). Barium titanate nanopowder, provided by Grafen Chemical Industries Co. (Ankara, Turkey), was used as an additive material. The properties of spherical barium titanate nanopowder are given in Table 1.

### 2.2. Methods

#### 2.2.1. Preparation of Polymer Solutions

The TPU polymer solution was prepared at a concentration of 16% in DMF solvent. The prepared mixture was magnetically stirred at 45 °C for 36 h. The BT powder was added to the prepared polymer solution at various rates (1, 3, 5, and 7% *w*/*w*) and then stirred by a mechanical homogenizer for 3 min at 10,000 rpm. The solutions were left to rest for 1 h to remove air bubbles. The composition of films is given in Table 2.

#### 2.2.2. Film Production by Casting Method

**Figure 1:**
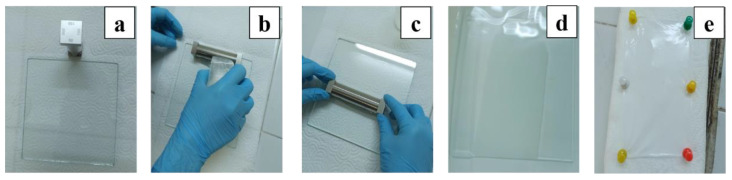
Solution casting process: (**a**) Film applicator; (**b**) Casting of the polymer solution; (**c**) Production of the film; (**d**) Removal of the solvent; (**e**) Drying of the film.

The films were made using a solution casting method with a manual film applicator (Figure 1a). The prepared solutions were poured onto the glass plate (Figure 1b), and then the applicator was pulled at a constant speed from one end of the plate to the other in one move (Figure 1c) so that the surface was covered with a wet film. The glass plate covered with wet film was immersed in pure water to remove the DMF (Figure 1d) and solidify the TPU. The solidified film was separated from the glass plate in the water. After removing the film from the water, the film was fixed on the foam plate with pins (Figure 1e) and dried for 24 h.

#### 2.2.3. Test and Characterization

The film thickness was measured using a digital thickness gage (Insize, Series 2871-101, Suzhou New District, China) with a sensitivity of ±0.005 mm. Measurements were taken on at least three different points on the films. SEM images of films with a gold coating at 10 kV acceleration voltage were taken with Carl Zeiss/Gemini 301 scanning electron microscope (Oberkochen, Germany) at a 100× and 10,000× magnification rate. FTIR spectra were obtained using the Shimadzu IR-Tracer100 (Kyoto, Japan) device equipped with an attenuated total reflectance (ATR) apparatus between 4000–400 cm^−1^ wavenumbers with a 4 cm^−1^ resolution by taking 32 scans. All spectra were collected under the condition of applying the same pressure to all samples. The differential scanning calorimeter (DSC 25, TA Instruments, New Castle, DE, USA) was utilized to determine the melting temperature and degree of crystallinity of pure thermoplastic polyurethane and its composite films loaded with varying concentrations of barium titanate nanoparticles. The heating thermograms were recorded under a nitrogen environment with a heating rate of 10 °C/min from 40 to 250 °C. The formula for calculating crystallinity is shown in Equation (1) [61],

(1) $$x_{c}\left(\%\right)=\frac{{∆H}_{m}}{{x\times ∆H}_{100}}\times 100$$

where x_c_ is the degree of crystallinity, x is the weight fraction of TPU in the composite material, ΔH_m_ represents the melting enthalpy, and ΔH_100_ is the melting enthalpy of 100% crystalline polymer (172.2 J/g for TPU) [62,63].

The TGA analyses were conducted using a Shimadzu DTG-60H TG/DTA simultaneous measuring instrument (Kyoto, Japan). TGA curves, the decomposition temperature ranges, and mass loss values were obtained. The initial and the final temperatures were 20 °C and 800 °C, respectively. The heating rate was 20 °C/min, and the gas type was nitrogen.

The Shimadzu UV-3600i Plus spectrophotometer (Kyoto, Japan) was used for transmittance, reflectance, and absorbance measurements of the samples in the wavelength range of 280–2500 nm, according to EN 14500 standard [64]. The solar transmittance (T_S_: 300–2500 nm), solar reflectance (R_S_: 300–2500 nm), near-infrared reflectance (R_NIR_: 800–2500 nm), and ultraviolet transmittance (T_UV_: 280–380 nm) values were obtained according to the EN 410 standard [65]. The opacity values of the film samples were determined using Equation (2) as described by Han and Floros [66],

(2) $$Opacity=A_{600}/X$$

where A_600_ is the absorbance at 600 nm and X is the film thickness (mm). 

The water contact angle measurements were determined using the sessile drop method under ambient conditions by KSV-The Modular CAM 200 Contact Angle Measurement System (Toronto, ON, Canada). The images were taken 3 s after the water droplet (9 μL volume) was applied to the film surface. The measurements were performed ten times for each sample. Tensile tests were conducted using a Shimadzu AG-X plus tensile tester (Kyoto, Japan). The dimensions of the sample strips were 10 × 100 mm. The tensile speed and gauge length were set at 100 mm/min and 80 mm, respectively. Samples were conditioned before all tests and analyses.

## 3. Results

### 3.1. SEM Image Analysis

**Figure 2:**
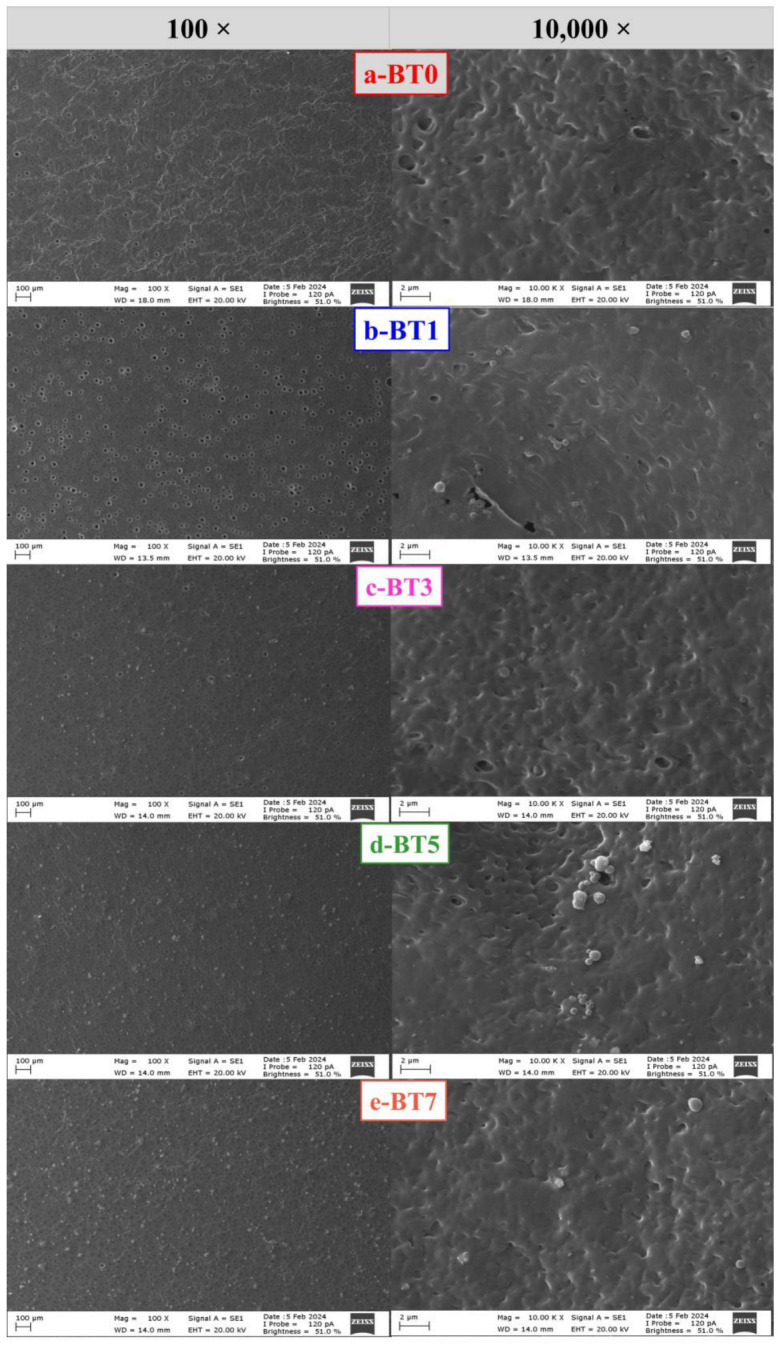
SEM micrographs of pure polyurethane film (**a**) and polyurethane films containing 1% (**b**), 3% (**c**), 5% (**d**), and 7% (**e**) barium titanate additive.

The morphology of pure TPU and nano-sized barium titanate-added TPU films were examined via SEM analysis. SEM photos belonging to the produced films are given in Figure 2. The SEM analyses clearly demonstrated that the production method of the solvent casting technique caused the formation of round holes resulting from the rapid removal of the solvent [67,68,69,70]. It was also seen that barium titanate powders were embedded in the pores and inside the TPU films. Figure 2 illustrates that with an increase in the amount of additive, particularly for BT5 and BT7, the nanoparticles became more visible on the surface, as expected. Even though nano-sized barium titanate powders were clustered (10,000×) on some parts of the film surfaces, there was a homogeneity in the overall distribution (100×). When the morphology of TPU films-added barium titanate was evaluated, it was observed that pore formation decreased with the increase of barium titanate addition in the structure. Similar structures in films obtained by the solution casting method and pore changes observed with increasing additives have been reported by other studies [71,72].

As a result, the obtained images in this study are compatible with the literature, and the addition of nano-sized barium titanate significantly affects film morphology.

### 3.2. FTIR Analysis Results

**Figure 3:**
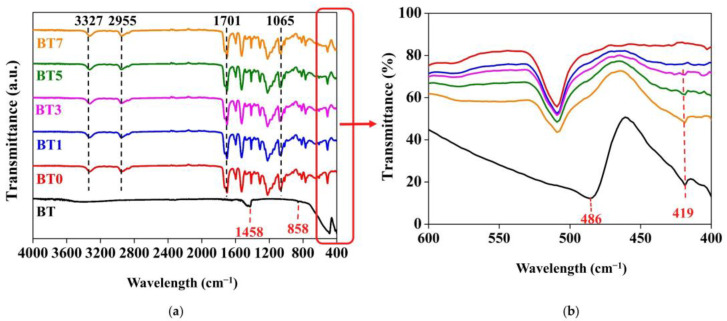
FTIR spectra of barium titanate powder (BT), pure polyurethane film (BT0), and barium titanate-added polyurethane films (BT1–BT7) at wavelength ranges of (**a**) 4000–400 cm^−1^ and (**b**) 600–400 cm^−1^.

The interaction between barium titanate and the TPU matrix was defined through the FTIR spectra, as presented in Figure 3. The pure TPU, the nano-sized barium titanate-added TPU films, and the barium titanate nanopowder (BT) were investigated in the region of 4000–400 cm^−1^. The pure TPU (BT0) spectrum exhibited absorption peaks at 3327 cm^−1^ and 1701 cm^−1^, linked to the stretching vibration of N-H and C=O bonds, respectively [73,74,75,76]. The signal at 2955 cm^−1^ corresponded to the stretching vibration of the C-H bond. In addition, the absorption at 1065 cm^−1^ was attributed to the C–O–C stretching vibration [73,77,78].

The absorption seen at approximately 419–486 cm^−1^ in the BT spectrum was associated with the vibrations of the Ti-O bond and it was a characteristic peak for barium titanate [79,80,81]. The peak located at 1458 cm^−1^ was an intrinsic signal [82] of barium titanate, and it was assigned to the stretching or bending vibrations of carboxylic groups, which could come from the polymers (such as barium acetate, barium carbonate, etc.) used in the synthesis of barium titanate [83,84,85,86]. The peak at 858 cm^−1^ corresponds to stretching vibrations of metal–oxygen [87].

The absorption peaks of barium titanate were so weak that they were undetectable compared to the TPU signals. Thus, there was no significant disparity between the spectra of pure TPU and the BT-added films. Similarly, in some studies examining composites made by combining polyurethane with various polymers, it was noted that the composite structures exhibited all the typical absorption peaks of pure polyurethane [75], and their absorption peaks and spectra were very similar to pure polyurethane [88]. Gong et al. [89] also stated that the FTIR spectra of unfilled polyvinylidene fluoride (PVDF) and BT/PVDF nanocomposites showed no major difference. Increasing the barium titanate additive ratio did not considerably affect the curves’ intensity of films, which is consistent with the literature [27,90]. However, in the range of 419–486 cm^−1^, the spectra of the BT/TPU films showed a closer resemblance to the barium titanate powder (BT) as the amount of additive increased. The peak at 419 cm^−1^, which belongs to barium titanate, was also observed in the BT3, BT5, and BT7 samples. 

### 3.3. DSC and TGA Results

**Figure 4:**
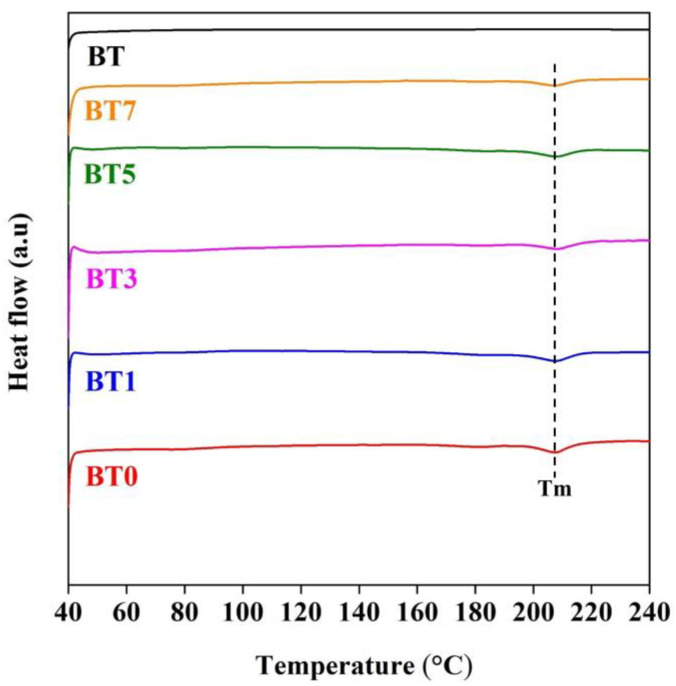
DSC curves of barium titanate powder (BT), pure polyurethane film (BT0), and barium titanate-added polyurethane films (BT1–BT7).

Figure 4 presents the DSC curves for BT nanoparticles, pure TPU, and composite films with added barium titanate, analyzed at temperatures from 40 to 240 °C. The barium titanate nanoparticles did not exhibit any melting peaks, as expected. Pure TPU and barium titanate-added composites gave melting peaks at approximately 207 °C. The increase in the barium titanate ratio did not significantly affect the melting temperature of the composite films, which is consistent with some studies in the literature. Mandal and Alam noted that the melting temperature of barium titanate-added poly(ether ether ketone) (PEEK) and poly(ether ketone) (PEK) composites did not change with an increasing additive ratio [91]. The study of Boschetto et al. also stated that increasing the barium titanate additive almost did not affect the melting temperature of PLA fibers [92]. Table 3 shows the heat of fusion (ΔH_m_) calculated from the DSC graph for composite films. The crystallinity (%) was calculated based on the area of the endothermic peak (Equation (1)). The crystallinity values of the additive films slightly increased compared to pure TPU film. In two studies using the PVDF matrix, it was observed that the degree of crystallinity increased in parallel with the rising barium titanate content in the composites [40,89].

**Figure 5:**
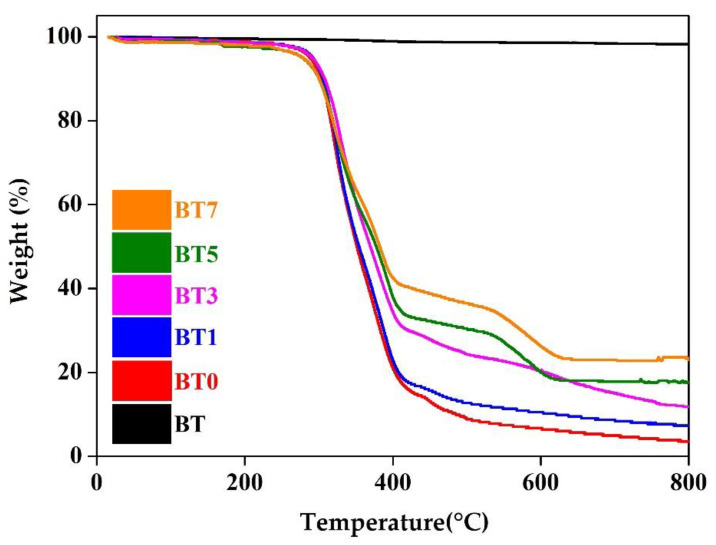
TGA curves of barium titanate powder (BT), pure polyurethane film (BT0), and barium titanate-added polyurethane films (BT1–BT7).

TGA curves of barium titanate nanoparticles, pure TPU, and barium titanate-added films are presented in Figure 5. The decomposition temperature ranges, total mass loss and residue values are also given in Table 4. The barium titanate nanoparticle (BT) retained approximately 99.0% of its weight with only a 1.02% loss, and half of this loss occurred in the 200–400 °C range. Similar to our results, two studies in the literature [40,93] reported that barium titanate nanoparticles were thermally stable up to 800 °C with only 1–2% weight loss, indicating their purity [40]. Also, in the study of Thanki et al., barium titanate samples exhibited stable curves in TGA analysis conducted in the range of 50–1000 °C [94]. 

Polyurethanes generally exhibit low thermal stability and may begin to decompose at temperatures exceeding 180 °C. The thermal decomposition of polyurethanes typically begins with hard segments, and various parameters such as soft segment and molecular weight significantly influence its thermal stability. Thermal stability, which mainly relies on the balance between the polymerization and depolymerization of functional groups in polymer molecules, can be enhanced using various inorganic additives [54,95]. In the present study, the pure TPU (BT0) remained stable up to 200 °C. Afterward, it exhibited a significant weight loss of approximately 72% in the 200–400 °C range, followed by a complete mass loss at around 650 °C. 

When analyzing the table data, it was observed that with increasing additive, mass losses decreased, and the residue amount increased due to the high thermal stability of barium titanate nanoparticles. The highest decomposition rates are observed in the 200–400 °C range. The film with the lowest amount of barium titanate additive (BT1) showed a total mass loss approximately 16.3% higher than the film with the highest amount of the additive (BT7). Similar to our results, in the study by Zhang et al., where they examined the impact of the filler loading level (1%, 5%, 8%, and 10%) on various properties of epoxy polymer nanocomposites (PNCs) filled with barium titanate, mass losses decreased with increasing filler ratio in PNC films [96]. The improvement in thermal stability of the composites is influenced by factors such as the type and quantity of inorganic filler, its dispersion within the matrix, and the extent of interaction between the filler and the matrix. In the literature, commonly, low amounts depending on the type of nanoparticle are seen as advantageous for high thermal stabilization. This is because nanoparticles are evenly distributed within polymer matrices, offering a high surface area of non-degradable material that helps protect macromolecular chains from thermal decomposition [95]. Xiong et al. noted that the inorganic additive can limit further degradation by preventing rapid heat dissipation in polyurethane composites [97]. Jana and Cho attributed the improvement in thermal stability to the fact that the inorganic additive may have acted as a thermal barrier in the first stage of degradation of polyurethane composites [98].

According to Table 4, the total mass loss of BT7 was 23.5% lower than that of pure TPU film. It was thought that the thermal stability of the composite films could be enhanced due to the increased char residue resulting from the interaction between barium titanate nanoparticles and the hard segments of the polyurethane [52]. The obtained results were similar to the examples in the literature. As one of these, Sharma et al. stated that the PVDF/BaTiO_3_ nanocomposite film gave a higher decomposition temperature than the pristine PVDF, thus improving thermal stability [40]. Bele et al., who examined the effects of adding different barium titanate fillers (commercial, cubic nanoparticles, and nanorods) to the polydimethylsiloxane (PDMS) film, revealed in the TGA analysis that the amount of residue substance (wt%) after 700 °C was higher in barium titanate-added films (especially for commercial filler) than in the pure crosslinked PDMS reference [99]. In support of this result, Yan et al., when they compared the TGA curves of polyaniline and polyaniline-coated barium titanate composite particles, stated that the amount of material remaining in the barium titanate-doped sample was higher [100]. 

### 3.4. Water Contact Angle Results

**Figure 6:**
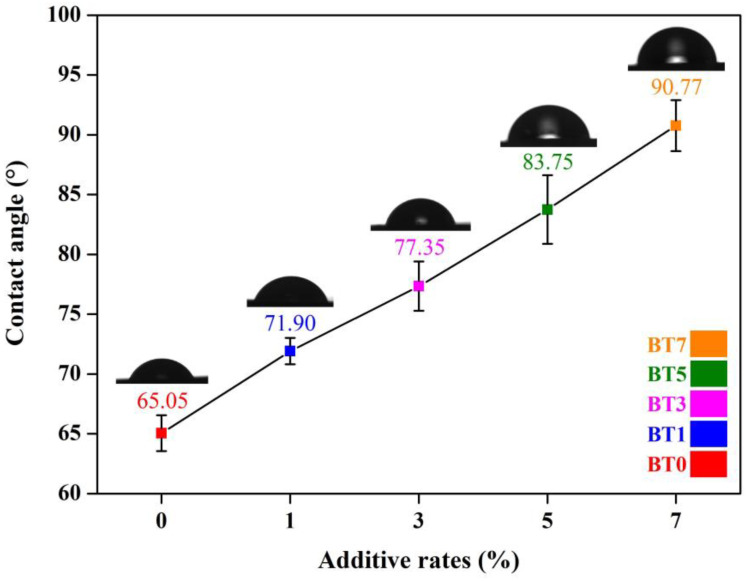
Contact angle values of pure polyurethane and barium titanate-added polyurethane films.

The contact angle is a critical parameter in material science [101]. It measures how a liquid droplet interacts with a solid surface. The contact angle provides a quantitative measure of a surface’s ability to be wetted by a liquid [102]. A low contact angle indicates high wettability, meaning the liquid spreads out more on the surface. Conversely, a high contact angle suggests low wettability, with the liquid forming a more rounded shape. Water contact angle values are influenced by the surface morphology, the chemical composition, the physical interactions among additives components, additives properties, and the production methods [103,104,105,106,107,108]. In this study, the contact angle values of the produced films with 1, 3, 5, and 7% additives were 71.90°, 77.35°, 83.75°, and 90.7°, respectively, as shown in Figure 6. 

While the contact angle value of the film surface without the additive was 65.05°, the contact angle value for the 1, 3, 5, and 7% additives increased by 10.5%, 18.9%, 28.7%, and 39.5%, respectively, compared to the film surface without the additive. Although the samples maintained their hydrophilic properties (θ < 90°) at the lowest barium titanate concentration, the repellency of water droplets on the film surface increased as the amount of barium titanate added to the film surface increased. At the highest additive rate, the contact angle value exceeded 90° and the surfaces showed hydrophobic properties. The incorporation of nanopowder changed the wettability towards hydrophobicity because it changed the pore formation properties as seen in SEM images. Furthermore, the high contact angle values observed for the additive films were attributed to the inorganic nature of barium titanate. The ATR-FTIR measurements, which, along with SEM analysis, confirmed the presence of BT particles both on the surface and within the polymer matrix. It was concluded that the presence of BT nanoparticles changing the surface morphology affected the wettability behavior. The increase in the contact angle value with the addition of barium titanate was also consistent with the literature. Due to the difference in the production method, the contact angle of the pure film surfaces was lower compared to the study of Şen et al., 2016. Despite this, the hydrophobic contact angle values could be achieved with the increase in the barium titanate added [51].

### 3.5. Solar Measurement and Opacity Results 

**Figure 7:**
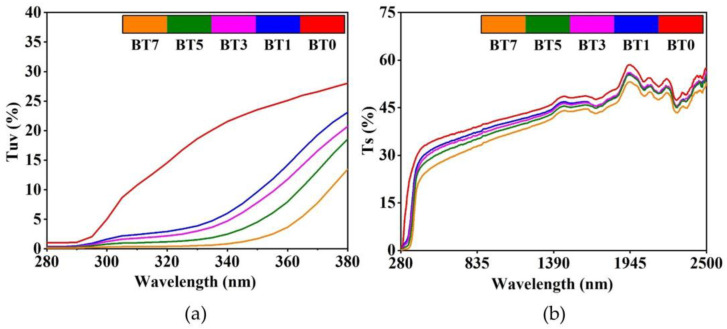
Transmittance spectra of polyurethane films added with barium titanate at different concentrations. (**a**) Ultraviolet region; (**b**) entire spectrum scanned.

**Figure 8:**
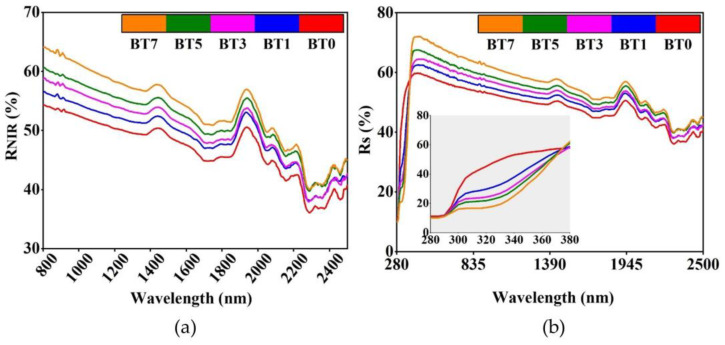
Reflectance spectra of polyurethane films added with barium titanate at different concentrations. (**a**) Near-infrared region; (**b**) ultraviolet region and entire spectrum scanned.

Table 5, Figure 7 and Figure 8 show the transmittance and reflectance values and spectra of the pure and barium titanate-added TPU films. When the solar transmittance values were analyzed (Figure 7b), it was noted that as the concentration of barium titanate increased, the Ts values decreased by 6.53%, 8.78%, 12.43%, and 19.20%, respectively, compared to the reference (BT0).

Contrary to the transmittance values, barium titanate addition increased the R_S_ values of TPU films by 3.59%, 6.74%, 10.26%, and 16.18%, respectively. Celen and Ulcay examined the solar properties of fabrics woven in different constructions from barium titanate-doped bicomponent polyester yarns. They stated that as the additive rate increased from 1% to 3%, transmittance values decreased in the entire scanned solar region, especially in the UV region, and solar reflectance increased [28]. In another study consistent with our results, Cai et al. stated that the transmittance values of the barium titanate films were decreasing with increasing solution concentration [109]. Celen et al. examined the solar properties of polyester fabrics coated with barium titanate at different concentrations. The UV transmittance (T_UV_) and the solar transmittance (T_S_) values decreased by 86% and 30.8%, respectively, compared to the reference at the maximum barium titanate concentration (100 g/kg) [27].

When examining the results in the near-infrared region (Figure 8a), the reflectance values increased by 3.97%, 7.45%, 10.75%, and 16.10% compared to the pure TPU film. In the study of Xiang and Zhang, barium titanate particles showed much higher reflectance in the VIS-NIR region compared with the neat acrylonitrile-styrene-acrylate copolymer (ASA) [30]. In two studies examining the near-infrared region, it was observed that barium titanate-doped samples gave higher reflectance values than their references [27,30]. 

**Figure 9:**
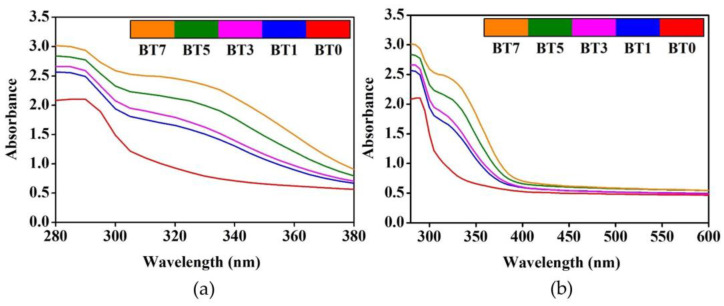
Absorbance spectra of polyurethane films added with barium titanate at different concentrations. (**a**) Ultraviolet region; (**b**) entire spectrum scanned.

Figure 7a shows that even at the lowest level of the barium titanate additive (BT1), T_UV_ was reduced by half compared to the pure polyurethane film (BT0). At the maximum additive rate (BT7), the value obtained is only 1/7 of the reference. Similar to transmittance, reflectance in the UV region was also lower in the barium titanate-added films compared to the pure reference (Figure 8b). This decrease is due to the strong UV absorbance property of the barium titanate particles [30,42]. The absorbance spectra seen in Figure 9 also confirmed this situation. In the ultraviolet region, there was a significant increase in absorbance compared to the reference, even at a 1% contribution (Figure 9a). This increase continued proportionally with the barium titanate amount. Chen et al. also stated that ultraviolet absorbance values increased as titania content increased in polyurethane/titania hybrid films [110].

Opacity is a measure used to describe transparency, and a higher opacity value indicates lower transparency [111,112]. It can also be expressed as the ratio of the light intensity falling on the material surface from a source to the amount of light transmitted. One of the parameters affecting the transmitted amount of light is the material composition [113]. As observed in Table 6 and Figure 9b, there was a slight but gradual rise in absorbance values at 600 nm as the barium titanate rate increased. The barium titanate additive slightly increased the opacity of pure polyurethane film. Adding 1% barium titanate to pure polyurethane film increased opacity by 17%, reaching 22% at the maximum additive rate (7%). 

In certain studies found in the literature, it was mentioned that adding nanoparticles to the polymer might lead to decreased transparency [114,115,116]. Although the trend toward increased opacity continued as the additive ratio increased, the opacity values of the additive films were generally similar to each other. Especially, the difference was almost negligible at 1% and 3% contribution rates (BT1 and BT3). At the 5% additive rate, the opacity only increased by 2% compared to 3%, while at 7%, the value increased by 2% compared to 5%. Some studies in the literature mention that nanoparticle additives dispersed in matrices can cause an increase in the crystallization degree of composites by serving as heterogeneous nucleating agents [89,117,118]. The crystallinity of polymer films significantly influences their transparency. Higher crystallinity enhances light scattering, which subsequently decreases transparency [119,120,121]. Based on this information, it was thought that the slight increases in opacity values in proportion to the nano barium titanate addition (Table 6) could be related and compatible with the increased crystallinity values (Table 3).

### 3.6. Tensile Strength Results 

**Figure 10:**
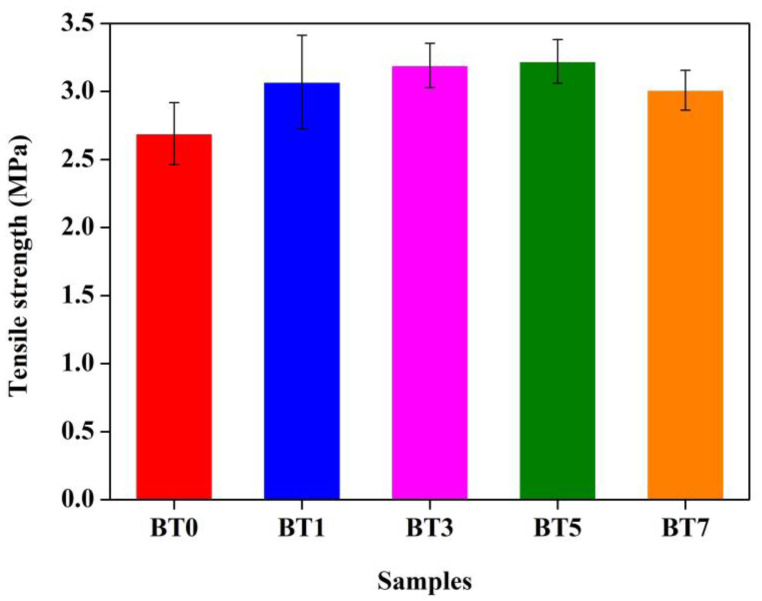
Tensile strength results of pure polyurethane and barium titanate-added polyurethane films.

In Figure 10, the strength test results of the samples are presented. The strength of the film produced with 1% additive did not significantly increase compared to the reference film (2.69 MPa). However, the strength values gradually increased with higher barium titanate amounts. The increases were 14.13%, 18.59%, 19.70%, and 11.90% for the BT1 (3.07 MPa), BT3 (3.19 MPa), BT5 (3.22 MPa), and BT7 (3.01 MPa) samples, respectively. Similarly, Zhang et al. indicated that the breaking stress results increased as the barium titanate nanoparticle addition increased [96]. Also, Guzman Sierra et al. stated that the tensile strength increased as the additive rate increased in samples containing varying amounts of barium titanate (0.6%, 1.2%, and 3%), and this demonstrated the reinforcing effect of barium titanate in the chitosan matrix [122]. Some other studies in the literature also indicated that the mechanical performance of the materials was improved with barium titanate addition [123,124]. Studies in the literature stated that exceeding a certain concentration of nanoparticles can reduce strength rather than improve it further [125,126]. In the current study, after the 5% additive rate, the upward trend continued compared to the pure TPU film, but the increase rate declined, and the value of the BT7 sample decreased to 3.01 MPa from 3.22 (BT5). As a result, it was observed that the addition of barium titanate enhanced the mechanical performance of the polyurethane film to some extent.

## 4. Conclusions

The objective of this study was to enhance the properties of polyurethane films by adding a barium titanate nanoparticle. FTIR and SEM analyses confirmed that barium titanate-added polyurethane films were successfully produced using the solution casting method. The addition of barium titanate, ranging from 1% to 7%, improved the thermal stability of the pure polyurethane film and reduced the total mass loss by approximately 24%. The melting temperatures of the composite films generally remained stable. The contact angle values of the TPU film, exhibiting hydrophilic properties, increased gradually with the barium titanate concentration. Upon adding 7% barium titanate, the wettability behavior changed, resulting in a hydrophobic surface. Due to the absorbance property developed with the barium titanate additive in the ultraviolet region, the transmittance values decreased dramatically, even at the lowest additive content. Although the films containing barium titanate exhibited slightly higher opacity values compared to the pure polyurethane film, their results were similar. The strength value of the polyurethane film was increased by up to 20%, depending on the additive ratio.

Polyurethane films containing barium titanate, known for its ecological aspects among ceramic materials, are expected to find use in various applications due to their improved thermal stability, hydrophobic properties, and potential for ultraviolet shielding.

## Figures and Tables

**Table 1:** Properties of barium titanate nanopowder.

Property	Value
Average particle size (nm)	100
Density (g/cm^3^)	5.85
Purity (%)	99.9
Surface area (m^2^/g)	10–11

**Table 2:** Composition of barium titanate-added polyurethane films.

Sample	DMF (mL)	TPU (g)	BT Nanopowder (g)	BT Nanopowder * (%)
BT0	10	1.6	0	0
BT1	10	1.6	0.016	1
BT3	10	1.6	0.048	3
BT5	10	1.6	0.080	5
BT7	10	1.6	0.112	7

* BT content relative to the initial mass of TPU.

**Table 3:** DSC data of films.

Sample	T_m_ (°C)	ΔH_m_ (J/g)	X_c_ (%)
BT0	207.74	5.75	3.34
BT1	207.78	5.66	3.38
BT3	208.45	6.16	3.68
BT5	207.78	6.43	3.93
BT7	207.31	7.41	4.63

**Table 4:** TGA data of films.

Sample	Temperature Range (°C)	Mass Loss (%)	Total Mass Loss (%)	Residue (%)
BT	200–400	0.51	1.02	
	400–650	0.17		98.98
	650–800	0.34		
BT0	200–400	71.69	98.45	
	400–650	24.63		1.55
	650–800	2.13		
BT1	200–400	75.55	91.27	
	400–650	13.53		8.73
	650–800	2.19		
BT3	200–400	64.13	86.77	
	400–650	17.23		13.23
	650–800	5.41		
BT5	200–400	59.69	80.11	
	400–650	19.90		19.89
	650–800	0.52		
BT7	200–400	55.34	75.00	
	400–650	19.66		25.00
	650–800	0.00		

**Table 5:** Solar measurement results (average ± SD).

Sample	T_UV_ %	T_S_ %	R_NIR_ %	R_S_ %
BT0	21.67 ± 1.92	37.82 ± 1.57	50.61 ± 1.64	54.56 ± 1.23
BT1	10.07 ± 0.99	35.35 ± 1.96	52.63 ± 0.79	56.52 ± 0.66
BT3	8.37 ± 1.01	34.50 ± 2.07	54.38 ± 1.49	58.24 ± 1.53
BT5	5.86 ± 0.75	33.12 ± 1.05	56.05 ± 1.34	60.16 ± 1.08
BT7	3.03 ± 0.52	30.56 ± 1.13	58.76 ± 0.53	63.39 ± 0.53

**Table 6:** Absorbance (at 600 nm), thickness, and opacity results of the films (average ± SD).

Sample	BT0	BT1	BT3	BT5	BT7
A_600_	0.464 ± 0.02	0.496 ± 0.02	0.506 ± 0.02	0.543 ± 0.02	0.546 ± 0.02
Thickness (mm)	0.086 ± 0.004	0.078 ± 0.003	0.080 ± 0.004	0.084 ± 0.003	0.084 ± 0.003
Opacity (A/mm)	5.43 ± 0.37	6.34 ± 0.47	6.36 ± 0.25	6.48 ± 0.23	6.61 ± 0.15

## Data Availability

The original contributions presented in the study are included in the article, further inquiries can be directed to the corresponding author.
